# The activation of lactate dehydrogenase induced by mTOR drives neoplastic change in breast epithelial cells

**DOI:** 10.1371/journal.pone.0202588

**Published:** 2018-08-23

**Authors:** Marcella Manerba, Lorenza Di Ianni, Marzia Govoni, Antonietta Comparone, Giuseppina Di Stefano

**Affiliations:** Department of Experimental, Diagnostic and Specialty Medicine (DIMES), University of Bologna, Bologna, Italy; University of South Alabama Mitchell Cancer Institute, UNITED STATES

## Abstract

mTOR kinase and the A isoform of lactate dehydrogenase (LDH-A) are key players controlling the metabolic characteristics of cancer cells. By using cultured human breast cells as a “metabolic tumor” model, we attempted to explore the correlation between these two factors. “Metabolic tumors” are defined as neoplastic conditions frequently associated with features of the metabolic syndrome, such as hyper-insulinemia and hyper-glycemia. MCF-7 cells (a well differentiated carcinoma) and MCF-10A cells (a widely used model for studying normal breast cell transformation) were used in this study. These cells were exposed to known factors triggering mTOR activation. In both treated cultures, we evaluated the link between mTOR kinase activity and the level of LDH expression / function. Furthermore, we elaborated the metabolic changes produced in cells by the mTOR-directed LDH-A up-regulation. Interestingly, we observed that in the non-neoplastic MCF-10A culture, mTOR-directed up-regulation of LDH-A was followed by a reprogramming of cell metabolism, which showed an increased dependence on glycolysis rather than on oxidative reactions. As a consequence, lactate production appeared to be enhanced and cells began to display increased self-renewal and clonogenic power: signals suggestive of neoplastic change. Enhanced clonogenicity of cells was abolished by rapamycin treatment, and furthermore heavily reduced by LDH enzymatic inhibition. These results highlighted a mechanistic link between metabolic alterations and tumorigenesis, whereby suggesting LDH inhibition as a possible chemo-preventive measure to target the metabolic alterations driving neoplastic change.

## Introduction

mTOR kinase regulates cell growth and proliferation in response to growth factors and nutrients [1]. It forms the catalytical subunit of two distinct complexes, known as TORC1 and TORC2. The molecular mechanisms regulating mTOR kinase are still poorly understood, although its constitutive activation has been repeatedly observed in cancer lesions [2]. Furthermore, negative regulation of mTOR by two Tuberous Sclerosis complexes (TSC1-2) was found to result in tumor suppression [3].

The TORC1 pathway induces cell growth by promoting protein synthesis [1]. Moreover, it promotes a shift in glucose metabolism from oxidative phosphorylation to glycolysis, which, as stated above, facilitates incorporation of nutrients into new biomass [4]. On the other hand, TORC2 complex is involved in the control of cell proliferation and survival and its direct target AKT has been found to regulate both glycolytic and oxidative metabolism [5].

Change in energy metabolism is one of the hallmarks of cancer cells and lactate dehydrogenase (LDH) is a key player in its orchestration [6]. The A isoform of LDH (the so-called “Warburg enzyme”) is constantly up-regulated in neoplastic tissues; by actively reducing pyruvate to lactate, LDH-A ensures rapid ATP production and oxidized NAD regeneration, both needed to support cancer cell proliferation. However, increased LDH-A activity also causes enhanced lactate generation, with its consequent export in the extracellular milieu. The metabolite diffusing from malignant cells stimulates hyaluronan synthesis in surrounding fibroblasts, causing a rearrangement of extra-cellular matrix, facilitating invasive cell growth [7]. Moreover, lactate was found to increase cancer cell migration by promoting matrix metalloproteinase-2 (MMP- 2) activity [8]. For these reasons, lactate levels in cancer tissues can be viewed as both a mirror and a motor of tumor malignancy [7]. In clinical studies, increased LDH-A levels have been found to be associated with poor prognosis in a variety of tumor forms [6, 9].

According to their role in cancer cell metabolism, mTOR and LDH-A could be expected to be functionally related. To the best of our knowledge, the only study directly examining the LDH / mTOR relationship concerned the B isoform of the enzyme (LDH-B) and was performed in TSC2 -/- murine embryonic fibroblasts, which display mTOR hyperactivation [10]. However, while LDH-A contribution in neoplastic change is widely rated, the impact of LDH-B in cancer cell biology is less defined. Furthermore, due to its different kinetics [6], LDH-B might not be expected to substantially raise lactate levels in tumors, although, as stated above, this compound seems to be a crucial link between cancer cell metabolism and tumor progression.

In the present work, we examined the LDH / mTOR relationship using a breast cancer cell model. The choice of this model found its justification from the data collected in several epidemiological studies, showing that the most common forms of breast cancer are usually associated with metabolic alterations, such as hyperglycemia, increased blood levels of insulin and obesity, so that they can be viewed as “metabolic tumors” [11].

Experiments were performed on MCF-7 and MCF-10A cell cultures. MCF-7 is a well-studied model reproducing the features of the metabolic breast cancer usually diagnosed in the post-menopausal female population (a well differentiated tumor, ER and PR positive). MCF-10A are non-neoplastic, spontaneously immortalized breast epithelial cells [12].

Both cultures have been exposed to known factors leading to mTOR activation and resulting changes in LDH expression and/or activity were verified. Since activation of mTOR kinase was found to have an impact on LDH-A expression and function, we studied the outcome of this effect on cell biology.

## Materials and methods

### Cell culture and reagents

MCF-10A and MCF-7 cells (ATCC-LGC Standards) were maintained in DMEM (5 mM glucose) supplemented with 10% FBS. 2 mM glutamine, 100 U/ml penicillin/streptomycin. Medium of MCF-10A cultures also contained 0.5 μg/ml hydrocortisone and 100 ng/ml cholera toxin. Experiments were always performed by maintaining the cultured cells at the physiologic glucose level (5 mM), to avoid forcing metabolism towards aerobic glycolysis. Prior to each experiment, cultures were maintained in serum deprived medium for 24 h. Cells were routinely screened for Mycoplasma contamination and found to be free.

Unless otherwise specified, all media and reagents used for the experiments were from Sigma-Aldrich.

### Immunoblotting detection

Cells were seeded in T25 flasks at a density of 2 x 10^4^ /cm^2^ and allowed to adhere overnight. After 24 h incubation in serum deprived medium, cultures were exposed to insulin (10 μg/ml), enhanced glucose level (20 mM) or leucin (1 mM) in serum deprived medium.

At different time intervals, cells were lysed in RIPA buffer supplemented with protease and phosphatase inhibitors. Homogenates were left 30 min on ice and then centrifuged 15 min at 10000 g. Proteins of the supernatants (20–30 μg, measured according to the method of Bradford) were loaded onto 4–12% pre-casted polyacrylamide gels (Life Technologies) for electrophoresis. The separated proteins were blotted on a low fluorescent PVDF membrane (GE Lifescience) using a standard apparatus for wet transfer with an electrical field of 80 mA. The blotted membranes were blocked with 5% BSA in TBS-Tween and probed with the primary antibodies: p70 S6 Kinase and phospho-p70 S6 Kinase; AKT and phospho-AKT (Ser473) (193H12); LDH-A and phospho-LDH-A (Tyr10) (all from Cell Signaling); c-MYC (from ABCAM).

Binding was revealed by a Cy5-labelled secondary antibody (anti rabbit-IgG, GE Lifescience; anti mouse-IgG, Jackson Immuno-Research). All incubation steps were performed according to the manufacturer’s instructions. Fluorescence of the blots was assayed with the Pharos FX scanner (BioRad) at a resolution of 100 μm, using the Quantity One software (BioRad).

For some experiments, after fluorescence detection the PVDF membranes were washed twice with TBS-Tween, stained with 0.1% Coomassie Blue R-350 dissolved in 50% methanol, de-stained with acetic acid / ethanol / water (1:5:4), rinsed with water and let to dry. Intensity of the protein bands migrated in the 20–60 kDa range (assessed with the Pharos FX scanner) was used to normalize the levels of the immunodetected proteins among the different samples, according to the method described by Weliner et al. [13].

### Real Time-PCR

Real Time-PCR was performed by following the procedure already described in [14]. Cells were seeded at a density of 2 x 10^4^ /cm^2^ and allowed to adhere overnight. After 24 h incubation in serum deprived medium, cultures were exposed to insulin (10 μg/ml) dissolved in serum deprived medium. For the inhibition of mTOR signaling, some cultures were exposed to both insulin and rapamycin (100–200 nM) for 24h. At different time intervals, RNA was extracted according to the procedure described by Chomczynski and Sacchi [15] and was quantified spectrophotometrically. Retro-transcription to cDNA was performed by using the Revert Aid TM First Strand cDNA Synthesis Kit, in different steps: 5 min denaturation at 65°C, 5 min annealing at 25°C, 1 h retro-transcription at 42°C and 5 min denaturation at 70°C. Real Time PCR analysis of cDNA was performed using SYBR Green (SSO Advanced, BioRad).

The primers used for analyzing expression of the studied proteins were as follows:

LDH-A: Forw: 5′-GACCTACGTGGCTTGGAAGA-3′, Rev: 5′-TCCATACAGGCACACTGGAA-3′;
LDH-B: Forw: 5′-CCAACCCAGTGGACATTCTT-3′, Rev: 5′-AAACACCTGCCACATTCACA-3′;
ALDH13A: Forw: 5′-TGGATCAACTGCTACAACGC-3′, Rev: 5′-CACTTCTGTGTATTCGGCCA-3′;
Ki67: Forw: 5′-GAGGTGTGCAGAAAATCCAAA-3′, Rev: 5′-CTGTCCCTATGACTTCTGGTTGT-3′;
OCT4, Forw: 5′-CGACCATCTGCCGCTTTG-3′, Rev: 5′-CCCCCTGTCCCCCATTCCTA-3′;

For all examined proteins, except OCT4, annealing temperature of primers was 60°C and the thermal cycler (CFX96 TM Real Time System, BioRad) was programmed as follows: 30 sec. at 95°C; 40 cycles of 15 sec. at 95°C; 30 sec. at 60°C. For the study of OCT4 expression, annealing temperature of primers was 56°C and the thermal cycler was programmed as follows: 30 sec. at 95°C; 40 cycles of 10 sec. at 95°C; 30 sec. at 56°C and 1 min at 72°C. Samples were run in triplicate, in 10 μl reaction volume containing 100 ng of cDNA.

To select adequate internal controls of the PCR reaction, expression of different genes, conventionally used as internal controls, was tested; beta2-microglobulin (beta2-M) and HPRT were found to be minimally affected by experimental conditions and used for sample normalization. The used primers were:

beta2-M: Forw: 5′-CATTCCTGAAGCTGACAGCATTC-3′, Rev: 5′-TGCTGGATGACGTGAGTAAACC-3′;
HPRT: Forw: 5′- AGACTTTGCTTTCCTTGGTCAGG-3′, Rev: 5′-GTCTGGCTTATATCCAACACTTCG-3′.

### Cell viability

Untreated cells and cells exposed to insulin (10 μg/ml, 24 h) were seeded in 96-multiwell plates (2 x 10^4^ cells/well) and allowed to adhere overnight. After a further 24 h incubation, cell growth was evaluated by applying the Neutral Red staining, using the following procedure. Cells were maintained 3 h at 37°C with the Neutral Red dye dissolved in medium at the final concentration of 30 μg/ml. Medium was then removed and the cells were solubilized with 200 μl of 1% acetic acid in 50% ethanol. Absorbance of the solutions was measured at lambda540 using a microplate reader (Multiskan Ascent FL, Labsystems). The same procedure was used to assess the effect of oxamate (0–60 mM) on the viability of untreated cultures and of cells exposed to insulin (10 μg/ml, 48 h).

### Assay of lactate levels

Untreated cells, cells exposed to insulin (10 μg/ml, 24 h) and cells exposed to insulin + 200 nM rapamycin were seeded in 6-multiwell plates (5 x 10^5^ cells/well) and allowed to adhere overnight. Medium was then discarded and cultures were maintained in Krebs-Ringer buffer for 3 h at 37°C. Produced lactate (released in medium + intracellular metabolite) was assessed by applying the method described by Farabegoli et al. [16]. The same procedure was used to assess the effect of oxamate (0–60 mM) on lactate levels of untreated cultures and of cells exposed to insulin (10 μg/ml, 48 h).

### Evaluation of oxygen consumption rate (OCR)

This assay was performed by using a phosphorescent oxygen-sensitive probe (MitoXpress probe) from Luxcel Biosciences, as previously described [16, 17]. Cells from each line (5 x 10^4^ / well) were seeded in four wells of a 96-multiwell clear bottom, black body plate and allowed to adhere overnight. After the addition of the MitoXpress phosphorescent oxygen-sensitive probe (10 pmoles/well), plate was placed in a VictorTM fluorescence reader (Perkin Elmer) at 30°C and was monitored for about 20 min to reach temperature and gas equilibrium and to obtain basal signals. The wells were then sealed with mineral oil and monitoring of the signal was resumed for the next 60 min. During this interval, the increase of fluorescence signal, which indicates oxygen consumption, was measured every 60 sec. with 340/642 nm excitation/emission filters, a delay time of 30 μsec and a measurement window of 100 μsec. All dispensing steps of the experiment were performed at 30°C with pre-warmed solutions. For each cell line, evaluation of respiration was performed by applying the linear regression analysis to the time profiles of fluorescence signals obtained from the four wells, in order to determine the slope of the profile, which indicates OCR. The Prism 5 GraphPad software was used. This assay was applied to untreated cells and to cultures exposed to insulin (10 μg/ml, 48 h).

### Assay of ATP levels

ATP levels were measured using the CellTiter-Glo Luminescent Cell Viability Assay from Promega, as described previously [16, 17]. For this experiment, 2 x 10^4^ cells in 200 μl of culture medium were seeded into each well of a 96-multiwell white body plate and allowed to adhere overnight. After incubation in the presence of scalar amounts of oxamate (0–60 mM), the plate was allowed to equilibrate at room temperature for 30 min and the CellTiter-Glo reactive was directly added to each well. The plate was kept on a shaker for 10 min to induce cell lysis and its luminescence was measured by using a Fluoroskan Ascent FL reader (Labsystems). This assay was applied to untreated cells and to cultures exposed to insulin (10 μg/ml, 48 h).

### Clonogenicity assay

It was performed as previously described in [14]. MCF-10A cells were plated into 6-well plates, at the density of 250 cells/well, in triplicate. Three wells received no treatment (control cultures). All other cultures (9 wells) were exposed to 10 μg/ml insulin for 48 h; in the following 24 h three wells were treated with 200 nM rapamycin and another three wells received 60 mM oxamate. Compounds were then removed and cultures were maintained for additional 15 days. Medium was changed every other day. Cells were stained with 0.5% crystal violet (dissolved in 6% glutaraldehyde). Visible colonies were counted to generate a histogram.

### Statistical analysis

All statistical analyses were performed using the software GraphPad Prism 5. Each experiment was repeated two or three times, with at least triplicate samples per treatment group. Results are expressed as mean ± SE of replicate values. P values < 0.05 were considered statistically significant.

## Results

### mTOR activation in cancer and immortalized normal cells

As a first step in our study, we induced mTOR activation by exposing both MCF-7 and MCF-10A cells to hormonal factors or nutrients. After 24 h incubation in serum-free media cells were probed with either 10 μg/ml insulin, 1 mM leucin or 20 mM glucose. For both cell lines, insulin response had been repeatedly documented in previous studies [18, 19]. Metabolic substrates and growth factors are well-recognized triggers of TORC1 pathway [1]. On the contrary, upstream signals of the TORC2 complex have been less extensively studied; the second mTOR complex is considered part of the PI3K-AKT pathway and is involved in response to growth factor signaling [5].

**Fig 1:**
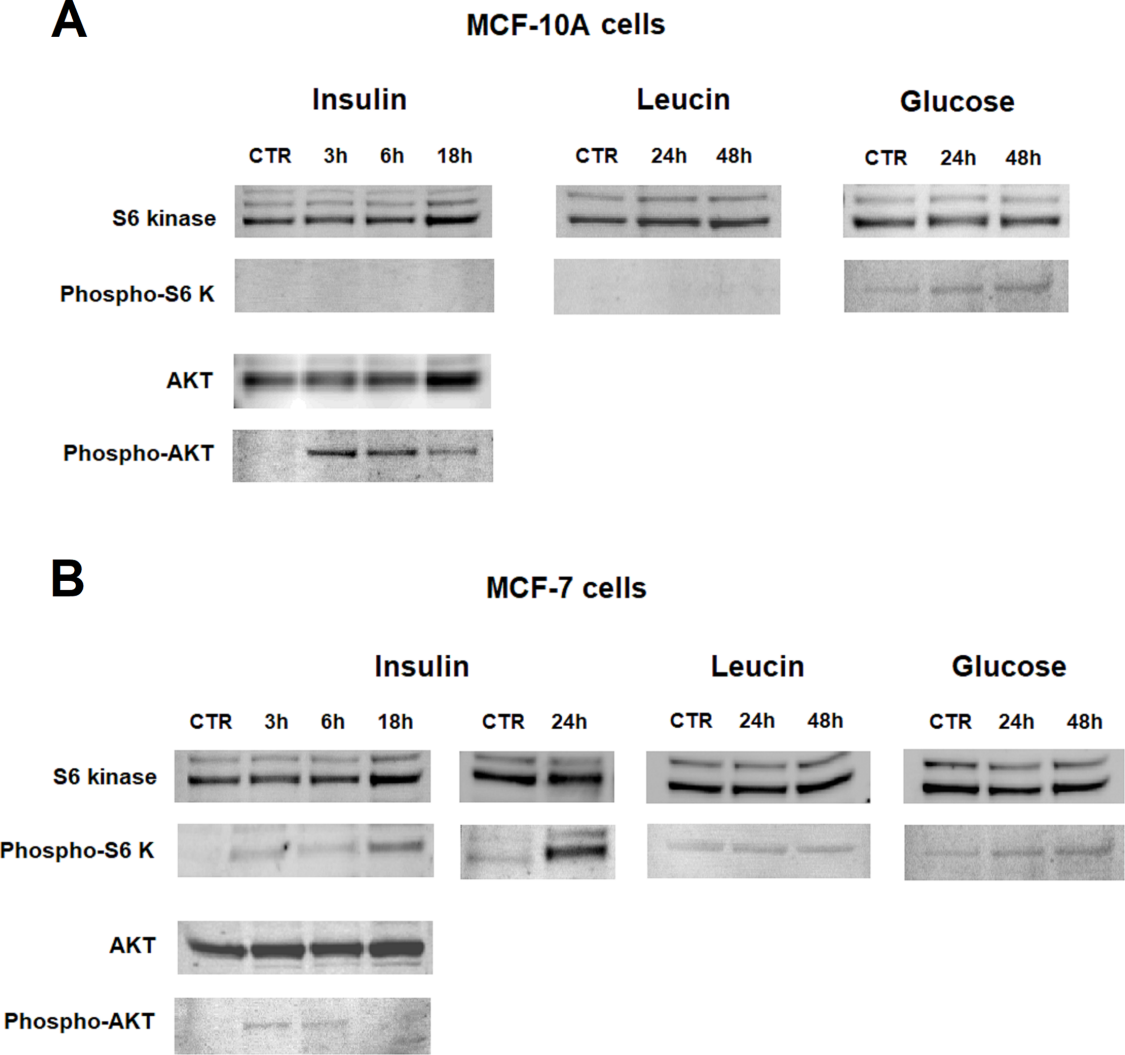
**Profile of mTOR activation observed in MCF-10A (A) and MCF-7 cells (B) exposed to 10** μ**g/ml insulin, 1 mM leucin or 20 mM glucose.** The experimental procedure was reported in Materials and Methods. For each time interval, the same sample (20 μg) was separately loaded onto two gels. After blotting, one of the PVDF membranes was probed with either the anti-S6K or anti-AKT antibody; the second membrane was probed with either the anti-phospho-S6K or the anti-phospho-AKT antibody. Activation of the mTOR pathways can be evaluated from the appearance of signals of protein phosphorylation. TORC2 pathway was found to be activated in MCF-10A cells; TORC1 pathway in MCF-7 cells.

In treated cells, mTOR activation was assessed by verifying the phosphorylation of S6K and AKT, the direct targets of TORC1 and TORC2 pathways, respectively [1]. As shown in Fig 1, MCF-10A and MCF-7 cells showed a different mTOR activation profile. Moreover, in agreement with the notion of a competitive relationship between TORC1 and –2 induction [1], in both cell lines a prevalent activation of a single enzymatic complex was observed. Insulin was the most efficient inducer. It caused a rapid activation of the TORC2 pathway in MCF-10A cells (Fig 1A), as indicated by the immunoblotting detection of phosphorylated AKT, which markedly appeared at 3–6 h and subsequently declined at 18 h. No sign of TORC1 activation was observed in these cells. On the contrary, in MCF-7 cells insulin challenge induced the phosphorylation of S6K, which appeared at 18 h and reached sharp evidence at 24 h (Fig 1B). Some minor activation of TORC2 was observed in these cells. In our experimental conditions, the exposure to metabolic substrates in high doses (1 mM leucin, 20 mM glucose) did not substantially induce mTOR kinase activation up to 48 h. For this reason, insulin challenge was used to investigate the mTOR / LDH relation in subsequent experiments.

### Activation of mTOR kinase results in increased LDH-A expression and activity

**Fig 2:**
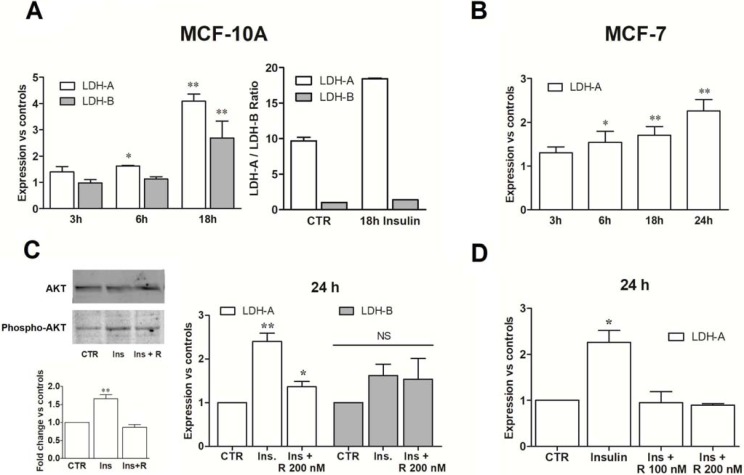
mTOR activation enhances LDH-A expression. Evaluation of LDH-A and LDH-B expression in MCF-10A (A) and MCF-7 cells (B) exposed to insulin. (C) In MCF-10A cells, rapamycin co-administration reduced AKT phosphorylation and LDH-A expression. (D) Rapamycin abolished the effects of insulin in MCF-7 cells. All results were statistically evaluated by ANOVA, followed by Tukey post-test. * and ** indicate a statistically significant difference compared to control cells with p < 0.05 and 0.01, respectively. (R, rapamycin).

Fig 2 shows changes in LDH-A and -B expression measured by real time PCR in MCF-10A (panel A) and MCF-7 cultures (panel B) challenged with insulin. Evaluations were performed at the time intervals indicated by the results reported in Fig 1. The levels of LDH mRNA of treated cultures were compared with those measured in control cells maintained in standard medium for the indicated time intervals after a 16 h serum starvation.

MCF-10A cells were found to express both the LDH isoforms. After a 18 h exposure to insulin a sharp increase of LDH-A mRNA was observed, which followed the peak of TORC2 activation reported at 3–6 h (see Fig 1A). A less marked, but statistically significant increase of LDH-B mRNA was also observed.

To assess whether the increased LDH expression observed in the experiments of Fig 2A could be attributed to mTOR activation, we had previously verified the effect of rapamycin on the activation of TORC2 pathway. Rapamycin is a well-known TORC1 inhibitor, which also showed activity on the TORC2 complex, especially on sustained treatments [20]. Fig 2C shows that a 24 h-exposure to rapamycin reduced AKT phosphorylation. At the same time, this compound almost completely abolished the increase of LDH-A expression when administered together with insulin (24 h). This experiment also showed that the mRNA level of LDH-B was not affected by rapamycin, suggesting that the increased expression observed in insulin-treated MCF-10A cells at 18 h is not related to mTOR activation. Furthermore, a comparison between the respective mRNAs levels (Fig 2A) showed that in MCF-10A, LDH-A mRNA is 10 to15-fold higher than that of LDH-B; for these reasons in subsequent experiments only LDH-A was considered.

Fig 2B shows that in MCF-7 cells exposed to insulin a progressive increase of LDH-A mRNA was observed, which paralleled the activation of TORC1 complex. Again, this increase was found to be completely reversed by rapamycin (Fig 2D). Contrary to MCF-10A cells, in MCF-7 cultures LDH-B mRNA was not detectable.

**Fig 3:**
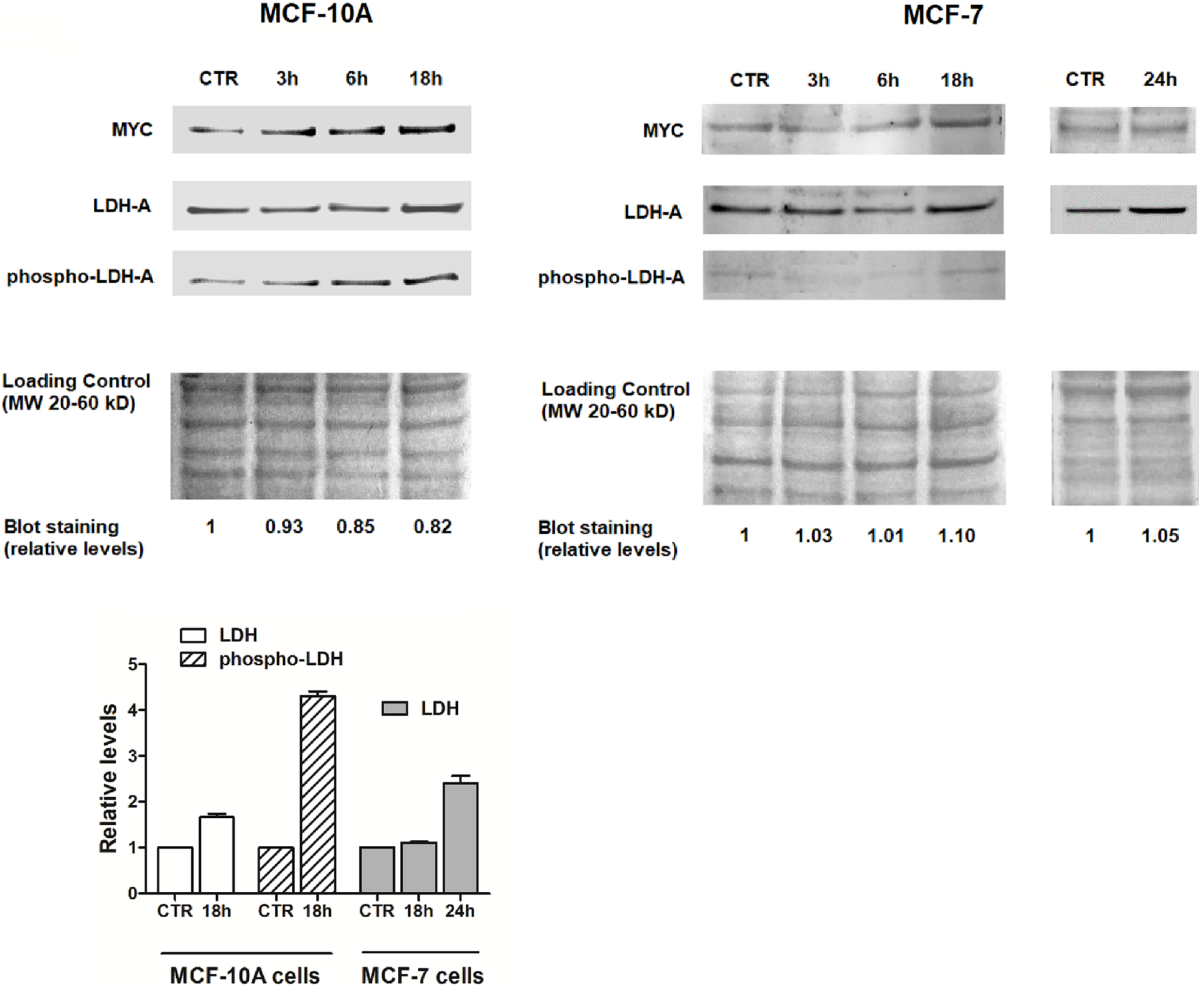
mTOR activation enhances LDH-A protein level and phosphorylation in MCF-10A cells. Evaluation of LDH-A and c-MYC protein levels in MCF-10A and MCF-7 cells at different times after insulin exposure. Activation of LDH-A through Tyr10 phosphorylation was also assessed. The Blue Coomassie staining of protein bands was used as loading control (for explanation, see text). The bar graph shows the quantification of relative levels of LDH-A and phospho-LDH-A in insulin-treated vs untreated cells.

In both cell cultures, increased transcription of LDH-A resulted in enhanced protein levels, as shown in the immunoblotting evaluations reported in Fig 3.

One of the major factors inducing LDH-A gene transcription is c-MYC. c-MYC is also a direct target of the TORC1 / S6K pathway [21] and is enhanced by TORC2 activation [22]. In agreement with these data, increased levels of MYC protein were detected in both cell cultures following insulin challenge (Fig 3), producing a mechanistic link between mTOR activation and the increased LDH-A levels.

**Fig 4:**
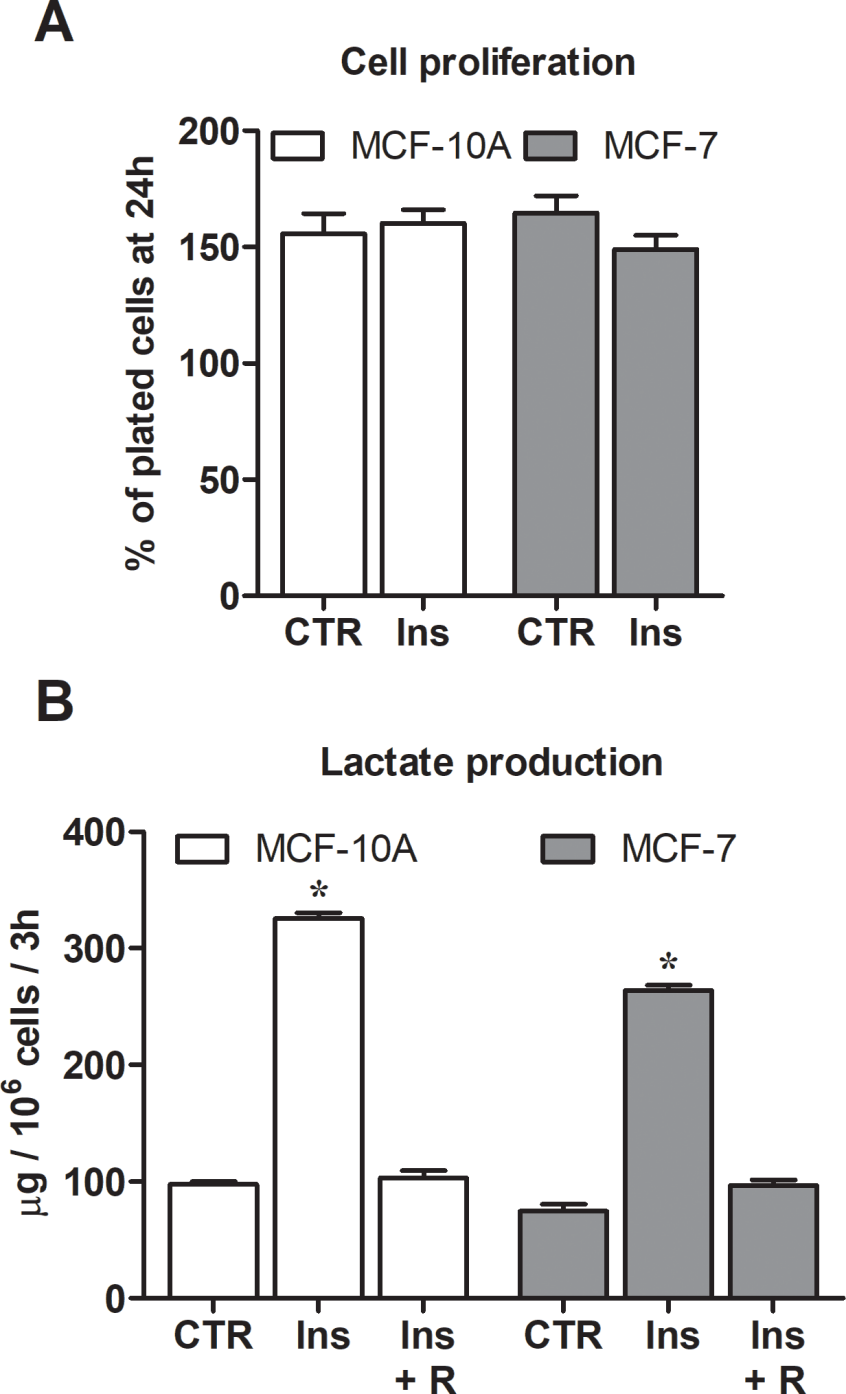
Effect of insulin on cell proliferation and lactate production. (A) MCF10-A and MCF-7 cell proliferation measured after 24 h exposure to insulin. (B) Lactate production rate was significantly increased by insulin (*, p < 0.01 compared to control cells); the increase was abolished by rapamycin co-treatment. Statistical analysis was performed by ANOVA followed by Tukey post-test. R, 200 nM rapamycin.

Remarkably, in MCF-10A a progressive increase of LDH-A phosphorylation on Tyr-10 was also observed. This post-translational modification has been repeatedly reported in cancer cells with over-activated LDH-A and, to the best of our knowledge, has never been detected in non-tumor tissues [23]. It can presumably contribute to the heavily increased enzymatic activity measured in MCF-10A cells after insulin challenge (see below and Fig 4B).

The bar graph of Fig 3 reports a semi-quantitative evaluation of the increased levels of LDH-A and phospho-LDH-A. Calculation was performed by using the densitometric evaluation of Coomassie-stained protein bands migrating in the 20–60 kDa molecular weight range as loading control of the blot membrane. This procedure is usually applied when treatment of cells is found to affect the level of structural proteins commonly used as internal controls in immunoblotting experiments. The method described by Weliner et al. [13] was utilized.

### mTOR-activated LDH-A causes metabolic changes in immortalized normal cells

Previous studies have indicated that insulin can stimulate the growth and proliferation of a variety of cells in culture [24]. This was not the case in our experimental conditions.

As shown in Fig 4A, neither MCF-10A nor MCF-7 cells displayed enhanced proliferative potential, when maintained for 24 h in the presence of increased insulin level but at the physiological glucose concentration (5 mM). In the case of MCF-10A cells, this was also confirmed by an evaluation of Ki-67 levels (see below).

Fig 4B shows that in both normal and cancer cells insulin challenge sharply enhanced the rate of lactate production. LDH-A increase and/or activation observed as a consequence of insulin exposure would account for this result; the finding that rapamycin co-treatment succeeded in abolishing the enhanced lactate production (Fig 4B) allowed us to link this metabolic change to mTOR activation.

**Fig 5:**
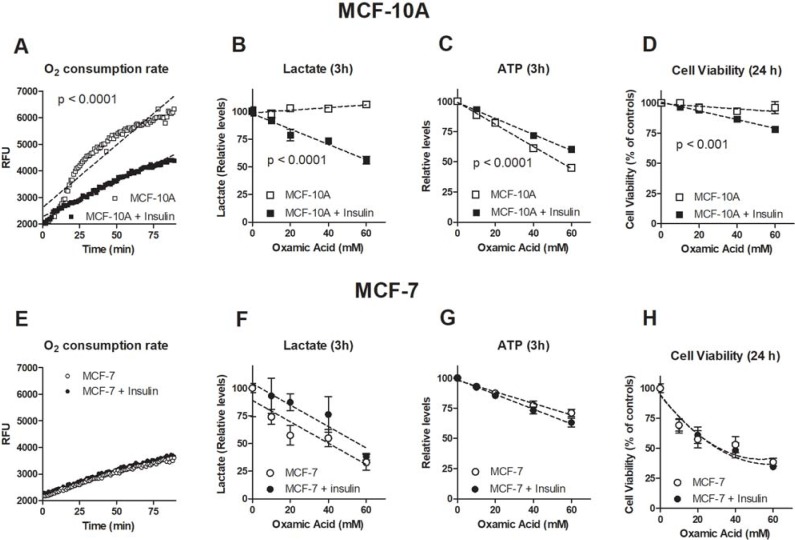
mTOR-driven LDH-A activation changes the metabolic features of MCF-10A cells. Metabolic parameters measured in MCF10-A (A-D) and MCF-7 cells (E-H) before and after a 48-h exposure to insulin. B-D and F-H graphs show the effect of OXA (a LDH inhibitor) on metabolic parameters. To evaluate the effect of insulin exposure on LDH inhibition, the experimental data were subjected to linear regression and the differences between curve slopes (data measured before *vs* data measured after insulin challenge, respectively) were statistically evaluated using the Prism 5 GraphPad software. A detailed explanation is reported in the text.

These results prompted a more detailed evaluation of the metabolic features induced by mTOR activation in both cell cultures. In control cultures and in insulin-challenged cells we evaluated the rate of oxygen consumption (OCR); moreover, to highlight possible changes in the metabolic asset after insulin treatment, we also studied the effect caused by oxamate on lactate and ATP levels and on cell proliferation. Oxamate is a pyruvate analogue which specifically inhibits LDH by competition with its natural substrate [25]. Results are reported in Fig 5. Upper panel (graphs A-D) show the results observed in MCF-10A cultures; results obtained in MCF-7 are reported in the lower panel (graphs E-H). To evaluate the differences in OCR and in response to OXA caused by insulin exposure, experimental data were subjected to linear regression. The differences between the curve slopes (before and after insulin treatment, respectively) were then evaluated by using the software Prism 5. Compared to MCF-7 cultures, untreated MCF-10A cells showed a markedly higher oxygen consumption, which is indicative of an energy metabolism predominantly based on oxidative reactions, as expected in non-tumor cells (Fig 5A and 5E). Remarkably, treatment with insulin significantly affected MCF-10A oxygen consumption (p < 0.001), which dropped to levels close to that of the neoplastic culture while it did not modify OCR of MCF-7 cells (Fig 5A and 5E). This finding suggested a mTOR/LDH induced change in the metabolic profile of MCF-10A culture, which was confirmed in the following experiments. Fig 5B shows that lactate levels of untreated MCF-10A cultures were not affected by oxamate, even when administered at high doses. This finding can be explained considering that in cells with functional oxidative energy metabolism, lactate is not simply a waste product but, after re-oxidation by the mitochondrial form of LDH [26], it can enter the TCA cycle, becoming an ATP source [27]. By inhibiting LDH, oxamate can affect both lactate production and utilization, whereby maintaining unchanged the level of this metabolite. Fig 5B also shows that after insulin exposure, oxamate caused a dose-dependent and statistically significant reduction of lactate production, as might be expected when energy metabolism has settled on aerobic glycolysis. These considerations also explain the findings reported in Fig 5C. By inhibiting lactate re-oxidation and its consequent utilization as energy source, oxamate caused a dose-dependent reduction of ATP levels in MCF-10A cells. This effect was found to be significantly reduced after insulin exposure, which hindered oxidative metabolism, the most efficient path of ATP generation. On the other hand, the metabolic impairment caused by the 3 h-incubation with OXA appeared to be overcome by MCF-10A cells at short times, since at 24 h their viability was not compromised by LDH inhibition (Fig 5D). These results are in agreement with the notion that LDH inhibition preferentially affects neoplastic tissues and is not toxic for normal cells [6]. Interestingly, insulin treatment was found to increase the effect of oxamate on MCF-10A cell viability, causing a moderate extent, but statistically significant reduction.

The findings reported in Fig 5F–5H show that insulin treatment did not significantly change oxamate effects in MCF-7 cells, both on lactate / ATP levels and on cell proliferation. These results suggest that when the glycolysis-based metabolic program has already been activated by neoplastic change, its further escalation is not easily evidenced, at least in the short observation time of our experimental settings.

By comparing the data reported in Fig 5A–5D with those in Fig 5E–5H, it can be concluded that in MCF-10A cells the reduced OCR observed after insulin exposure led to a reprogramming of cell metabolic asset. This is evidenced by the increased response to LDH inhibition, which appeared to acquire a similar profile as that observed in MCF-7 cells. Taken altogether, these results suggested that insulin-activated mTOR/LDH-A axis can significantly impact on MCF-10A cell physiology, inducing a metabolic profile close to that commonly observed in transformed, neoplastic cells.

### LDH-A-linked metabolic changes induce stem signatures, which are reversed by the enzyme inhibition

Since the metabolic features of cancer cells have repeatedly found to be linked with their infiltrative growth and with neoplastic progression [28], it seems reasonable to hypothesize that stimulation of the mTOR/LDH-A axis in non-tumor cells can result in a significant step towards neoplastic change.

This hypothesis was tested by searching in insulin-challenged MCF-10A cells features suggestive of a tumor-like phenotype. Increased capacity of self-renewal, clonal evolution and tumor initiating properties are conventionally indicated by the expression of stem markers [29]. Moreover, it is well known, that the presence of cells with stem-like properties is also indicative of worse prognosis in a variety of tumor diseases, including breast cancer [30, 31].

**Fig 6:**
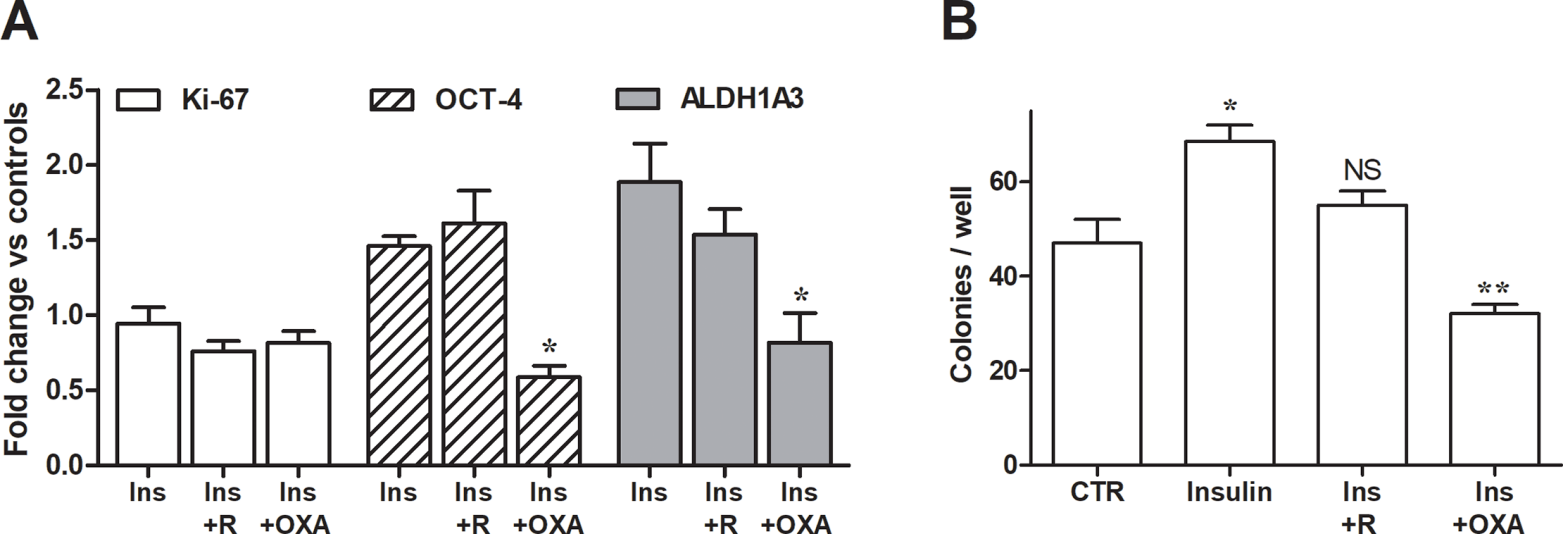
mTOR-driven LDH-A activation causes signs of tumor progression in MCF-10A cells. (A) Expression of Ki67, OCT4 and ALDH1A3 evaluated in MCF-10A cells after 48 h exposure to insulin. * indicates a statistically significant difference with insulin treated cells with p < 0.05. (B) Colony formation assay. Statistical analysis was performed by ANOVA followed by Tukey post-test. * indicates a statistically significant difference with control cells, with p < 0.05. ** indicates a statistically significant difference with insulin treated cells, with p < 0.01. NS, no significant difference compared to control cells. R, 200 nM rapamycin; OXA, 60 mM oxamate.

In breast neoplastic tissues, possible interconversion of cells from a stem to non-stem status has been observed [32]; furthermore, breast cancer stem cells were found to differentiate into different cell phenotypes. Regarding the MCF-10A line, previous studies have shown that these cells exhibit a basal-like phenotype while sharing many properties of mesenchymal cancer cell lines [33]. This aspect raised difficulties for the identification of reliable markers suggestive of the stem phenotype, since stem status cells often also express mesenchymal transition signatures [34]. In our experiments, the expression of Ki-67, OCT-4 and ALDH1A3 was analyzed in control and insulin-treated MCF-10A cells. Ki-67 is a well-established marker of proliferative potential [35]. OCT-4 is a homeodomain transcription factor with a key role in cell self-renewal and pluripotency [36]; its expression was found to be under the control of LDH-A [37]. ALDH1A3 is the ALDH isoform predominantly detected in breast cancer stem cells [38]. In patients, it was found to significantly correlate with tumor grade, metastasis, and cancer stage [39]. Furthermore, an assay to evaluate cell clonogenic potential was performed. Results are reported in Fig 6.

As shown in Fig 6A, insulin treatment did not affect the level of Ki-67 expression; this result is in agreement with the data reported in Fig 4A, which had already showed an un-changed proliferation rate in MCF-10A cells exposed to insulin. On the contrary, mTOR/LDH-A activation appeared to affect both OCT-4 and ALDH1A3 expression, which were increased by 50% and 100%, respectively. Remarkably, rapamycin co-treatment did not succeed in controlling the insulin-induced effects, which, on the contrary, were significantly reversed by oxamate. Consistent results were also obtained by applying the clonogenic assay (Fig 6B). A 48-h exposure to insulin was found to significantly increase the number of colonies formed by MCF-10A cells; this increase was abolished by the administration of rapamycin (200 nM, 24 h), since cells treated with this inhibitor showed a clonogenic potential superimposable to that of control, untreated cells. Inhibition of LDH (60 mM oxamate, 16 h) caused a sharp decrease of formed colonies (p< 0.01, compared to insulin treated cultures).

These results suggest that, although ineffective on the proliferative capacity of MCF-10A cells, the metabolic changes induced by mTOR/LDH activation had a significant impact on their biology, causing the enhanced manifestation of features indicative of neoplastic potential. These features appeared to be reverted by a reprogramming of cell metabolism obtained by LDH inhibition. This finding is in agreement with previous data showing that breast cancer stem cells are dependent on glycolysis for their survival [40, 41].

## Discussion

Constitutive activation of mTOR kinase is constantly observed in neoplastic tissues and its contribution to cancer cell biology has been extensively studied [2]. However, in spite of the crucial role of this kinase in metabolic regulation, its possible relationship with LDH has not been clearly demonstrated. The A isoform of LDH is the so-called “Warburg enzyme”; it is constantly up-regulated in neoplastic tissues and confers to cancer cells peculiar metabolic features which are considered one of the hallmarks of tumorigenesis [6]. The experiments reported in this paper showed that both the functionally active complexes TORC1 and TORC2 converge on LDH-A, by increasing its expression and/or activation. Our data showed that both LDH-A increase and its derived metabolic changes were reversed by rapamycin, the most studied mTOR inhibitor. Published results also showed that mTOR pathway was inhibited by glycolysis inhibitors [42, 43]. Taken altogether, these data suggest a reciprocal functional relationship between mTOR and LDH-A.

Interestingly, our results showed that induction of mTOR/LDH-A did not cause evident effects on the metabolic asset of MCF-7 cells, which because of their neoplastic nature already expressed the glycolysis-based metabolic program. On the contrary, the non-tumor immortalized MCF-10A cells appeared to be more susceptible to the effects of mTOR/LDH-A activation and underwent a metabolic reprogramming resulting in the manifestation of increased self-renewal and clonogenic potential. Previous data confirmed that breast cancer cells maintained in the presence of increased lactate levels could change their transcriptional profile, exhibiting increased progression markers [44]. Interestingly, in a variety of tumor forms lactate production is a recognized predictor of poor prognosis and lactate acidosis was found to be associated with breast cancer metastasis [7, 45]. By applying these results to our experimental model, it can be hypothesized that the 300% increased lactate levels measured in MCF-10A cells after mTOR/LDH-A activation (Fig 4B) could have affected gene expression, causing the appearance of the observed “stemness” signatures. The reduced capacity of using this metabolite as energy substrate because of the lower OCR of activated cells (Fig 5A) could have promoted this phenomenon.

These observations also imply that the strong dependence on glycolysis, which characterizes the energy metabolism of tumors, is not necessarily linked to gene mutations, but might also be induced by epigenetic mechanisms.

Our results appear to be significant, particularly in the light of the used cellular model. The MCF10A human mammary epithelial cell line is a widely used in vitro model for studying normal breast cell function and transformation. It was derived from a fibrocystic lesion [12], a tissue change very commonly observed in the female population, for which a possible association with increased cancer risk was debated.

Our experiments highlight a possible mechanism linking metabolic alterations such as hyper-insulinemia and/or hyper-glycemia to the induction of neoplastic change, confirming the association repeatedly observed in the epidemiological studies. Furthermore, our results are in agreement with progressively emerging data suggesting the possibility of epigenetic changes linked to metabolic factors, a notion underlying the concept of “metabolo-genomics”.

Currently, a number of therapeutic alternatives are available for curing breast cancer; a substantial advance in the fight against this disease could also come from a new approach, aimed at targeting through chemo-preventive procedures the metabolic alterations driving the neoplastic change. LDH inhibitors, which are not toxic for normal tissues [6], could be good candidates to test the validity of this approach.
